# Environmental Risk of Pesticides for Fish in Small- and Medium-Sized Streams of Switzerland

**DOI:** 10.3390/toxics9040079

**Published:** 2021-04-08

**Authors:** Inge Werner, Anke Schneeweiss, Helmut Segner, Marion Junghans

**Affiliations:** 1 Swiss Centre for Applied Ecotoxicology, 8600 Dübendorf, Switzerland; marion.junghans@oekotoxzentrum.ch; 2 Institute for Environmental Sciences, University Koblenz-Landau, 76829 Landau, Germany; schneeweiss@uni-landau.de; 3 Center for Fish and Wildlife Health, University of Bern, 3012 Bern, Switzerland; helmut.segner@vetsuisse.unibe.ch

**Keywords:** environmental risk assessment, insecticides, fungicides, herbicides, sublethal toxicity, resident fish species

## Abstract

This study assessed the acute and chronic risk of pesticides, singly and as mixtures, for fish using comprehensive chemical data of four monitoring studies conducted in small- and medium-sized streams of Switzerland between 2012 and 2018. Pesticides were ranked based on single substance risk quotients and relative contribution to mixture risk. Concentrations of the pyrethroid insecticides, λ-cyhalothrin, cypermethrin and deltamethrin, and the fungicides, carbendazim and fenpropimorph, posed acute or chronic single substance risks. Risk quotients of eighteen additional pesticides were equal or greater than 0.1, and thirteen of those contributed ≥30% to mixture risk. Relatively few substances dominated the mixture risk in most water samples, with chronic and acute maximum cumulative ratios never exceeding 5 and 7, respectively. A literature review of toxicity data showed that concentrations of several pesticides detected in Swiss streams were sufficient to cause direct sublethal effects on fish in laboratory studies. Based on the results of our study, we conclude that pesticides detected in Swiss streams, especially pyrethroid insecticides, fungicides and pesticide mixtures, pose a risk to fish health and can cause direct sublethal effects at environmental concentrations. Sensitive life stages of species with highly specialized life history traits may be particularly vulnerable; however, the lack of toxicity data for non-model species currently prevents a conclusive assessment across species.

## 1. Introduction

Contamination of surface water bodies with pesticides has been reported around the world [1,2] and attributed primarily to agricultural and, to a lesser extent, urban sources [3]. In Switzerland, several long-term monitoring studies conducted between 2012 and 2018 detected high numbers and concentrations of pesticides in small- and medium-sized streams predominantly flowing through agricultural land [4,5,6]. Between 100 and 145 pesticides were detected (corresponding to 40–67% of analytes), with 19–32 substances exceeding acute or chronic risk thresholds. On average, samples contained mixtures of 30–50 pesticides. In addition, analyses of pyrethroid insecticides (previously not analyzed) in 2017 and 2018 showed that these substances were also present at concentrations exceeding acute and chronic risk thresholds for aquatic organisms [7,8]. Most at risk were aquatic plants and invertebrates, and at times adverse effects on aquatic vertebrates, which includes fish and amphibians, could not be excluded [9,10]. Uncertainties with respect to the risk for fish remain, both with respect to the identification of the most fish-relevant pesticides, mixture risk effects and the potential for toxic effects on fish at environmental concentrations.

Regulatory environmental risk assessment of chemical water contaminants is performed by comparing exposure concentrations (predicted or measured) to predicted no effect concentrations (PNECs) in prospective risk assessment, or environmental quality criteria/standards in retrospective risk assessment. Both types of threshold values are derived by the same method using reliable and relevant ecotoxicity data (i.e., apical effects such as mortality, reduced growth and reproduction), which can be linked to population level effects [11]. Toxicity data for the most sensitive species, typically determined in standard toxicity tests, are identified and subsequently divided by an assessment factor (AF) to determine the risk threshold of each substance. The resulting risk assessment is considered to be protective of all species. In general, invertebrate or plant species are more sensitive to pesticides than fish. However, there are certain pesticides that are toxic to fish at equally or lower concentrations than other organism groups, e.g., ergosterole inhibiting fungicides [12,13].

The standard risk assessment approach largely ignores non-apical sublethal as well as indirect and multiple stressor effects of pesticides despite the fact that environmental concentrations of pesticides are often below lethal concentrations. Several reviews describe that pesticide concentrations clearly below LC50 levels can disrupt physiological, behavioral and hematological parameters in fish, such as anti-oxidant defense systems or energy metabolism. In addition, they can interfere with the neurological and immune systems and cause histopathological changes in barrier organs such as the intestine and gills and in internal organs such as liver and kidney [14,15,16,17,18,19]. Similarly, mixture effects are rarely taken into account despite their importance under environmental conditions [20,21,22]. Finally, adverse effects of pesticides to fish may arise not only from direct toxic effects but also through density- and trait-mediated effects [23,24]. Therefore, the risk of pesticides to aquatic organisms in the field may be severely underestimated [25]. Unexpected sublethal effects and the cumulative effects of contaminant mixtures and multiple stressors may, thus, reduce individual fitness and negatively affect populations of non-target species, potentially disrupting complex ecological systems (e.g., [26,27]).

This study focused on assessing the risk of pesticides for fish based on monitoring data from Switzerland, where 75% of the 55 indigenous fish species known are currently listed as regionally extinct, critically endangered, endangered, vulnerable or near threatened [28]. Among others, physical habitat changes, diseases as well as exposure to chemical contaminants have been named as potential causes [26]. Fish have complex life cycles and behaviors, can live for multiple years, and many species reproduce only once a year. Such traits may render them more vulnerable to chronic and sublethal toxic effects than the fractional spawners, which are typically used in laboratory toxicity tests, especially if exposure to stressors coincides with the presence of sensitive life stages. To gain a better understanding of the vulnerability of indigenous fish to pesticides detected in Swiss surface waters, (i) fish-specific risk thresholds were derived for pesticides detected in Swiss monitoring campaigns, (ii) pesticide concentrations measured in several recent monitoring studies were evaluated using a standard environmental risk assessment (ERA) to identify pesticides of highest concern for fish by ranking them according to single substance risk quotients and their relative contribution to mixture risk, and (iii) available non-apical effect data on fish-relevant pesticides were reviewed and discussed. In addition, we attempted to identify indigenous fish species potentially vulnerable to pesticides based on an established vulnerability concept.

## 2. Materials and Methods

### 2.1. Analytical Data and Sampling Design

Monitoring data on pesticide concentrations in Swiss streams for the years 2012, 2015, 2017 and 2018 are publicly available (SI, Table S1). Detailed information on sampling sites, water sampling and analytical methods is provided by [4,5,6] and for pyrethroids by [7,8]. In 2012, sampling sites were located in five medium-sized streams with catchment areas of 38–105 km^2^ comprising agricultural as well as urban areas. In 2015 and 2017, sampling sites were located in five small streams with catchment areas of 1.6–9 and 0.9–6.7 km^2^, respectively, dominated by agricultural land use. In 2017, pyrethroid insecticides were analyzed at one site only (Chrümlisbach, Canton Bern). In 2018, six sites in streams with catchment areas of 1.4–34 km^2^ were sampled and analyzed exclusively for 23 pyrethroids and the organophosphates (OP), chlorpyrifos and chlorpyrifos-methyl. At two sites (Eschelisbach, Canton Thurgau; Weierbach, Canton Basel-Land), samples were collected in both 2015 and 2017; Chrümlisbach (Canton Bern) and Bainoz (Canton Fribourg) were sampled in both 2017 and 2018.

Sampling designs of the four studies varied slightly. In 2012, 14-day time proportional (TP) composite samples were collected March to July and analyzed for 249 agricultural pesticides. In 2015, 12 h TP composite samples were collected March to August and analyzed for 213 agricultural pesticides. During dry periods between discharge events, 12 h samples were pooled corresponding to the length of the low flow period (5 d on average) and then analyzed. In 2017, 3.5-day TP composite samples were collected March to October and analyzed for 217 pesticides. In 2018, only 14-day TP composite samples were collected March to September, and chemical analysis focused on pyrethroids and OPs.

### 2.2. Risk Assessment—Single Substances

Fish-specific acute and chronic predicted no effect concentrations (PNEC_i,fish,acute_, PNEC_i,fish,chronic_) were derived for each pesticide detected and registered for use in Switzerland at the time of sampling, if sufficient toxicity data were available. Data were obtained from existing data collections of the Swiss Centre for Applied Ecotoxicology (Dübendorf, Switzerland; www.ecotoxcentre.ch), already evaluated for reliability and relevance according to CRED [11]. Additional data were compiled from the literature, primarily from approval reports of the European Food Safety Authority (EFSA, Parma, Italy; https://www.efsa.europa.eu), biocide assessment reports of the European Chemicals Agency (ECHA, Helsinki, Finland; https://echa.europa.eu/de/information-on-chemicals/), draft PNEC dossiers of the European Commission (EC, Ispra, Italy; https://ec.europa.eu/food/plant/pesticides/) and the US EPA OPP Pesticide Ecotoxicity Database (https://ecotox.ipmcenters.org/index.cfm?menuid=7) (SI, Tables S2 and S3). For the derivation of PNEC_i,fish,acute_, the concentrations that caused 50% mortality (LC_50_) within 96 h in the most sensitive fish species were divided by an assessment factor (AF) of 10. For the derivation of PNEC_i,fish,chronic_, concentrations that caused sublethal toxicity within ≥ 21 d (in most cases) the no observed effect concentration (NOEC) of the most sensitive fish species was divided by an AF of 10. This AF was chosen based on the European Union Guideline for the Derivation of Environmental Quality Standards (EQS) [29], where 10 is used as AF when acute and chronic toxicity data for all trophic levels, including the most sensitive, are available. If chronic data are missing, higher AF are used to extrapolate from acute to chronic toxicity. Because only fish data were used in this study and acute as well as chronic data were available, an AF of 10 was considered appropriate.

Fish-specific single substance risk quotients (RQ_i,acute_ and RQ_i,chronic_) were calculated by dividing the measured environmental concentrations (MEC) of a substance by the respective PNEC_fish_. If RQ ≥ 1, adverse acute or chronic effects of this substance on fish cannot be excluded. For the calculation of RQ_i,acute_, PNEC_i,fish,acute_ were compared to concentrations measured in acute exposure scenarios. Because sampling designs differed between studies, we used MECs of the smallest available collection period to determine acute risk. In 2015, collection periods for TP composite samples ranged from 0.5-day (wet periods) to 24-day TP composite samples (dry periods with assumed absence of exposure peaks). In this case, the PNEC_i,fish,acute_ was compared to the MEC of each sample analyzed irrespective of the collection period. For 2017 data, RQ_i,acute_ were determined using MEC of 3.5-day TP composite samples based on [30]. For 2015 and 2017 data, we used 14-day time-weighted (TW) average MEC for the calculation of RQ_i,chronic_. Acute risk was not determined for data generated in 2012 and 2018 when only 14-day TP composite samples were collected. For these samples, only the RQ_i,chronic_ was calculated.

### 2.3. Risk Assessment—Mixtures

Mixture risk quotients (RQ_mix,acute_ and RQ_mix,chronic_) were calculated by adding the RQ_i_ of all substances detected in the same water sample following current mixture risk assessment approaches [31]. Values below the limit of quantification (LOQ) were substituted with zero. If RQ_mix_ ≥ 1, adverse effects of the pesticide mixture on fish cannot be excluded.

### 2.4. Identification of Fish-Relevant Pesticides

In order to identify the most relevant pesticides to fish in Switzerland, results of the single substance and mixture risk assessments were used to rank individual pesticides. The primary selection criterion for relevance was RQ_i_ ≥ 0.1. It was chosen because fewer than 10 substances dominated the acute mixture risk based on PNEC_i,fish,acute_, even though the median number of substances present per water sample ranged from 23 to 42 [6]. If RQ_i_ ≥ 0.1 in at least in one water sample and the contribution to RQ_mix_ of ≥30% in the same sample, a substance was classified as highly relevant. The threshold of ≥30% contribution to mixture risk was considered representative based on the analysis of maximum cumulative ratios (MCR = ∑RQ_i_/RQ_i_,_max_), which indicates how many substances are drivers of the mixture risk [32]. In our dataset, the MCR ranged from 1–7 (acute) and 1–5 (chronic) (SI, Figure S1). To identify the most relevant pesticides, we chose a conservative MCR of 3 (equivalent to ca. 30% contribution per substance) as our secondary relevance criterion. These criteria were also applied to 2018 data, although analytes comprised only a limited set of insecticides, 23 pyrethroids and 2 OPs, chlorpyrifos and chlorpyrifos-methyl.

### 2.5. Review of Non-Apical Sublethal Effect Data

Publicly available data on sublethal effects were reviewed for fish-relevant substances during the summer of 2018 using Web of Science and Google Scholar with the following search terms: [compound], [endpoint or effect] and [fish].

### 2.6. Vulnerability of Indigenous Fish Species

Our approach to assess the vulnerability of fish species indigenous to Switzerland was based on the principle of vulnerability conceptualized by [33] and applied by [34]. First, we identified species particularly at risk of exposure to pesticides (“external exposure”). The occurrence of fish in habitats most likely to contain pesticides at concentrations of concern was determined with the help of Swiss fish monitoring data recorded by the National Surface Water Quality Observation program (NAWA TREND) [35]. Subsequently, we evaluated the “intrinsic species sensitivity” by ranking available acute toxicity data (LC_50_) for different fish species using species sensitivity distributions (SSDs), if reliable data were available for at least 5 species [36]. This was the case for the fungicides azoxystrobin and carbendazim, the insecticides chlorpyrifos, diazinon, cypermethrin and λ-cyhalothrin, as well as the herbicides linuron and diuron. SSD graphs were produced using R 3.5.3 [37].

## 3. Results and Discussion

### 3.1. Single Substance Risk

The fish-specific risk assessment revealed a total of 23 relevant pesticides with RQi ≥ 0.1. Table 1 provides a summary of results on these pesticides and their risk levels in different monitoring years, as well as the number of sites and days (across monitoring years) where exceedances occurred. All but five of these pesticides were classified as highly relevant, i.e., they contributed at least 30% to the mixture risk of the respective sample. Detailed information is shown in SI Table S4.

Concentrations of five pesticides (λ-cyhalothrin, cypermethrin, deltamethrin, carbendazim, fenpropimorph) exceeded their respective PNEC (i.e., RQ_i_ ≥ 1) at least once and, thus, posed a risk as individual substances. Among insecticides, the pyrethroid λ-cyhalothrin posed the highest risk in 2017 with an RQ_i_ ≥ 1 for both acute and chronic exposures. In 2018 (when data were limited to 14-day average concentrations and, thus, only chronic risk was assessed), two other pyrethroids, cypermethrin and deltamethrin, were detected at concentrations exceeding the chronic risk threshold (RQ_i,chronic_ ≥ 1). However, the 14-day MEC of deltamethrin (77 ng/L) not only exceeded the PNEC_i,fish,chronic_ by a factor of 45 (SI, Table S4), but also its acute risk threshold (PNEC_i,fish,acute_ = 15 ng/L) by a factor of 5 (SI, Table S4). In long-term laboratory studies, the 14-day MEC of this substance caused apical sublethal effects in fish, such as reduced growth (SI, Table S6). Based on runoff models for pyrethroids [38,39,40], it is possible, however, that one or more short (<1 day) pulses of very high deltamethrin concentrations occurred during the sampling period in 2018. Such brief peak concentrations of pesticides may greatly exceed concentrations detected in 3.5-day composite samples [41]. It is, therefore, possible that deltamethrin exceeded acute effect concentrations for fish, which demonstrates the severe environmental risk this substance might pose.

Besides the three pyrethroids, two fungicides, carbendazim and fenpropimorph, posed single substance risks in 2017 (carbendazim: acute and chronic, fenpropimorph: chronic). Although carbendazim was already banned in Switzerland in 2016, it was permitted for use until 2018, which explains its presence in water samples. Both fungicides were also detected at lower concentrations in previous monitoring studies (fenpropimorph in 2012, carbendazim in 2015) at multiple sites. In June 2017, tissue samples of fish (Leuciscus cephalus, a European cyprinid species) collected from the river Urtenen (BE) contained fenpropimorph (c_max_ = 20 µg/kg; Tamara Diethelm, WWF Schweiz, Zurich, Switzerland; personal communication). The river Urtenen is geographically close to Chrümlisbach, the monitoring site where high water concentrations of this compound were detected in 2017. Overall, the pattern seen suggests that water contamination by these two fungicides was widespread.

Another eighteen pesticides, among them four insecticides, nine fungicides and five herbicides, exceeded the fish-specific RQ_i_ (chronic or acute) of 0.1, most of them with regard to chronic risk. All insecticides were categorized as highly relevant. The OP insecticide, chlorpyrifos, which was not measured in 2012, exceeded both the acute and chronic RQ_i_ of 0.1 in 2015 and 2017 and the chronic RQ_i_ of 0.1 in 2018 at multiple sampling sites. As of May 2021, both chlorpyrifos and chlorpyrifos-methyl are no longer authorized for use in the EU and Switzerland (https://eur-lex.europa.eu/legal-content/EN/TXT/PDF/; https://www.psm.admin.ch/de/wirkstoffe/bs/C, both accessed on 28 January 2021). Another OP insecticide, diazinon, banned in Switzerland since 2011 [42], had a chronic RQ_i_ ≥ 0.1 in 2012 and 2015, but not anymore in 2017. Among five herbicides categorized as relevant based on chronic risk, four were highly relevant. S-metolachlor was detected at RQ_i_ ≥ 0.1 during a considerable part of the monitoring period, for 126 days in 2012 and 56 days in 2015, but not in 2017. Linuron was detected at chronic RQ_i_ ≥ 0.1 in all three years monitored, while diuron and aclonifen were detected at this level in 2015 only.

The risk of different pesticides determined for fish in Swiss streams varied from year to year, which is primarily a consequence of differences in application rates, weather conditions and changes in substance authorization status. Another important factor is the analytical capability and capacity. Monitoring and risk assessment results of pyrethroid insecticides in 2017 and 2018 highlight the importance of reducing so-called “blind spots” in the analytical data. In order to monitor this group of extremely toxic, hydrophobic insecticides, it is necessary to have analytical methods with limits of quantification (LOQ) in the picogram per liter range [7,33]. Once pyrethroid data became available in Switzerland, four pyrethroid insecticides were categorized as highly fish-relevant contaminants in our study, and all but one (permethrin) posed a single substance risk.

### 3.2. Mixture Risk

**Figure 1:**
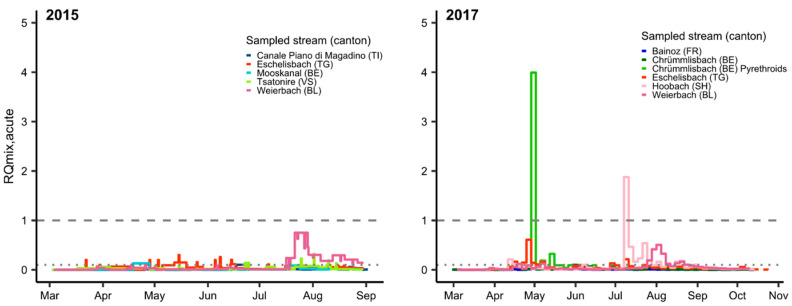
Acute mixture risk quotients (RQ_mix, acute_) for fish in composite water samples from small streams in Switzerland in 2015 and 2017. Two thousand and fifteen: acute exposure scenario (ExpSc) based on 0.5–24 day time-proportional (TP) composite samples; 2017: acute ExpSc based on 3.5 day TP composite samples; pyrethroids were analyzed at only 1 of 5 sites (Chrümlisbach, BE). RQ_mix_ (Chrümlisbach, BE, pyrethroids) is based solely on data for pyrethroids. Gaps indicate periods when no samples were collected; the dashed line indicates RQ = 1, and the dotted line indicates RQ = 0.1.

**Figure 2:**
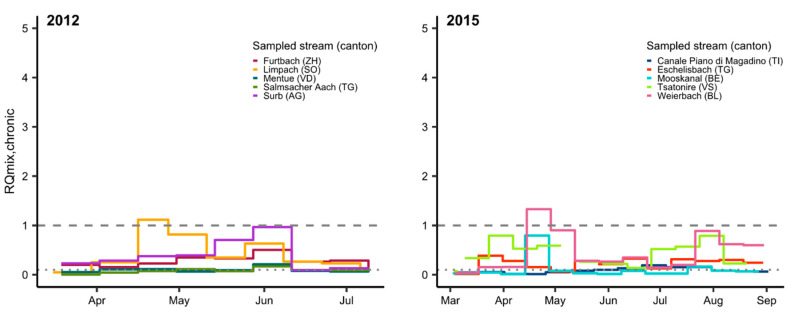

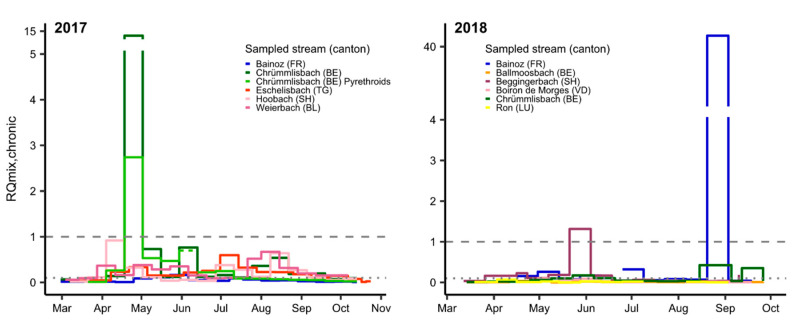
Chronic mixture risk quotient (RQ_mix, chronic_) for fish in water samples from small- and mid-size streams in Switzerland in 2012–2018. Two thousand and twelve and 2018: chronic ExpSc based on 14 day TP composite samples (except 16–27 April 2012: 11 day); in 2017 (Chrümlisbach, BE Pyrethroids) and 2018, RQ_mix_ is based solely on data for pyrethroids and chlorpyrifos/-methyl. Two thousand and fifteen and 2017: chronic ExpSc based on 14 day time-weighted average concentrations. Gaps indicate periods when no samples were taken; the dashed line indicates RQ = 1, and the dotted line indicates RQ = 0.1.

Pesticide mixtures presented a chronic or acute risk for fish at one or more sampling sites in each monitoring year, primarily in late spring and late summer (Figure 1 and Figure 2; SI Table S4). The acute mixture risk level for fish was exceeded on two occasions in 2017 (Figure 1). The peak in May was driven by the pyrethroid λ-cyhalothrin (>99%, in green), while carbendazim was the main driver (99%) in mid-July 2017.

Relatively few substances dominated the mixture risk in most water samples, with the chronic and acute MCRs never exceeding 5 and 7, respectively (SI, Figure S1). The fungicide fenpropimorph was responsible for 84% of RQ_mix,chronic_ in April 2012 (Limpach, SO). The pesticides responsible for most of the chronic risk seen in June 2012 (Surb, AG) were the fungicides fenpropimorph (35%), epoxiconazole (14%) and flusilazole (10%) and the herbicide S-metolachlor (17%). The herbicides, S-metolachlor (67%) and linuron (23%), dominated the chronic mixture risk in late April 2015 (Weierbach, BL).

Analyses of water samples collected from Chrümlisbach (BE) in late April/early May 2017 (pyrethroids and chlorpyrifos/-methyl analyzed separately [6,7]) revealed that the pyrethroid λ-cyhalothrin (91% of RQ_mix,chronic_ = 2.7) and the fungicide fenpropimorph (96% of RQ_mix,chronic_ = 13.1) were largely responsible for the high chronic risk. This was the only site where pyrethroid insecticides were analyzed in 2017. In 2018, monitoring of six sites was focused exclusively on pyrethroid insecticides, chlorpyrifos and chlorpyrifos-methyl. The chronic risk seen in late May/early June at Beggingerbach (SH) was driven almost entirely by cypermethrin (96%). In late summer, a peak in deltamethrin concentrations (99.8%) presented a very high risk to fish (acute and chronic) at Le Bainoz (FR). These results show that it is extremely important to monitor pyrethroid insecticides in surface waters. If not, the risk analysis would miss some of the most important environmental toxicants for fish as well as aquatic invertebrates.

### 3.3. Sublethal Effects

Major uncertainties exist in risk assessment with regard to the significance of the long-term, low-level chronic mixture risk (RQ ≥ 0.1 and <1) as observed at most sampling sites in this study (Figure 2). Between early March and mid-October 2017, for example, chronic mixture risk was elevated for >100 days at four of five monitoring sites, interspersed by short periods of acute or chronic risk ≥1. Information on the effects of such long-term exposures to pesticides is extremely scarce.

Sublethal effects can compromise individual fitness and ultimately lead to a decline of fish abundance [19,43]; however, conventional approaches to environmental risk assessment generally ignore data on non-apical sublethal effects. The risk to resident organisms exposed to low concentrations of pesticides for long periods of time may, therefore, be underestimated. Such effects include cellular effects resulting from the primary modes of action (MOA) of a pesticide or (often unknown) side effects. For pesticides categorized as highly fish-relevant in this study (marked * in Table 1), such primary MOA include the inhibition of cellular processes and components, i.e., sterole biosynthesis, respiration, inhibition of acetyl cholinesterase (AChE) and photosystem II, and the modulation of ion channels (SI, Table S5). Many of these cellular functions are conserved across organism groups; however, even herbicides and fungicides, which primarily target cellular mechanisms only present in plants or fungi, can affect fish via mechanism unrelated to their primary MOA [44]. Our analysis shows that neurotoxic insecticides, primarily pyrethroids, are of highest concern with regard to fish health. This finding corroborates conclusions of previous analyses [18,45,46]. Aside from affecting the nervous system of fish, pyrethroids have been shown to act as endocrine disruptors [47], genotoxicants [48] and immunotoxicants [49].

**Figure 3:**
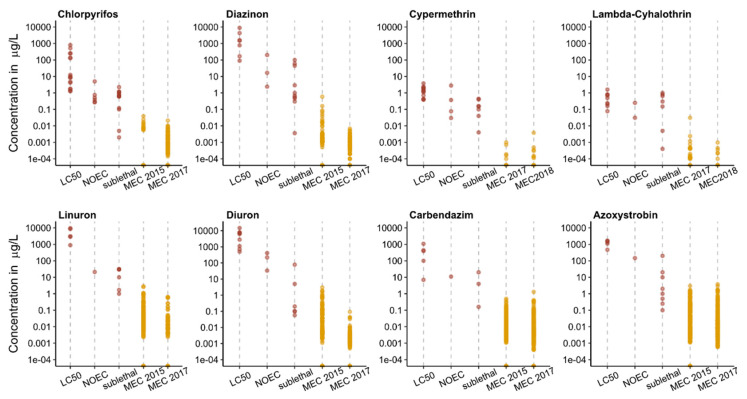
Comparison of LC_50′_s, no observed effect concentrations for apical sublethal endpoints (NOEC) and selected non-apical sublethal effect concentrations (sublethal) in red, with maximum environmental concentrations (MEC; in orange) measured in Switzerland in 2015 (0.5–24 day TP composite samples, [5]), 2017 (3.5 day TP composite samples, [6,7]) and 2018 (14 day TP composite sample, [7]). Values < LOQ were substituted with zero; 2017 MEC for chlorpyrifos from [7]. References are provided in SI, Table S7.

Figure 3 provides an overview of how MEC detected in Switzerland compared to regulatory toxicity thresholds (LC_50_, NOEC) as well as non-apical sublethal effect concentrations at or below the MEC of selected fish-relevant pesticides. While known LC_50′_s for fish are generally well above MECs, maximum pesticide concentrations detected in Switzerland have been reported to cause non-apical sublethal effects in fish under laboratory conditions (SI Table S6 and references therein). It is important to note, however, that most reported chronic and sublethal effect data are derived from test exposures longer than 3.5 d. Nevertheless, sublethal effects can already occur after brief exposures to environmentally relevant pesticide concentrations (SI, Table S6 and references therein). For example, the MEC of λ-cyhalothrin (31 ng/L in a 3.5 d composite sample) detected in 2017 exceeded the concentration that caused DNA damage in mosquitofish (*Gambusia affinis*) exposed for 2 d [50]. Similarly, the organophosphate insecticides, chlorpyrifos and diazinon, affected cellular homeostasis, olfactory function and behavior of fish after a few days of exposure at MECs detected in Swiss creeks [51,52,53,54]. The herbicide, linuron, can induce stress in brown trout exposed for 4 d at environmental concentrations [55]. Brief (24 h) exposure to a relatively low concentration (5 µg/L) of diuron negatively affected the grouping behavior of goldfish [56], a response important for predator avoidance. The fungicide, azoxystrobin, was shown to induce stress-responsive genes of zebrafish at or below concentrations detected in Swiss rivers within a few days [57]. Carbendazim affected locomotor behavior of zebrafish larvae after a 5 d exposure to 0.16 µg/L [58]. Exposed larvae moved more slowly than the control group, suggesting that their feeding success and predator avoidance capability might be impacted. Such non-apical sublethal effects may compromise individual fitness and ultimately lead to a decline of fish abundance, for example, through disruption of olfactory and reproductive functions, as well as impairment of their swimming and feeding ability [19].

### 3.4. Vulnerability of Resident Fish Species

Small- and medium-sized streams are important spawning and rearing habitats for fish. While the data on indigenous fish species and their distribution in Switzerland are still incomplete, 47 are currently known to be resident in Switzerland [28]. Of those, approximately 25 species inhabit small- and medium-sized streams either permanently, periodically or sporadically [35] and are, thus, potentially exposed to pesticides. This includes species that are potentially threatened (e.g., brown trout (*Salmo trutta*)) or endangered (e.g., lake trout (*Salmo trutta*)) (SI, Table S9). Compilation of toxicity data for several data-rich pesticides identified as fish-relevant in our study showed that several model species commonly used in standardized tests (*Pimephales promelas*, *Oryzias latipes*, *Danio rerio*) tend to have moderate to low sensitivity, while salmonids and non-model species tend to be more sensitive (up to >100-fold) to such compounds (SI, Figure S2).

Taxonomy, life history and life stage can modify fish responses to environmental stressors, and specialist species are less likely to show tolerance stressors than generalists [59,60,61,62]. Species that reproduce once a year, and whose early life stages (ELS) hatch and rear during a period when highest pesticide risk occurs (April–May and August–September, Figure 1 and Figure 2) may be most vulnerable, because the population cannot compensate by reproducing during times when risk from pesticides is low [63,64,65]. Fish ELS are often most sensitive to chemicals [66]. In addition, larval and juvenile fish tend to be more specialized with regard to their prey [67,68], which puts them at greater risk of indirect effects in case their invertebrate prey species are depleted due to pesticides [27,69]. Among such species are brown trout who spawn from November to January, and embryos develop slowly to hatch in spring [70,71], and spring spawners with short developmental periods, such as common nase (*Chondrostoma nasus*), the salmonid, grayling (*Thymallus thymallus*), as well as two endangered species, stroemer (*Leuciscus souffia*) and spirlin (*Alburnoides bipunctatus*). The results of our vulnerability analysis are, however, based on few data and must be considered descriptive.

### 3.5. Uncertainties and Limitations of Our Risk Assessment

Sampling designs, analytes and analytical methods, as well as availability of toxicological data are important variables influencing the outcome of the risk assessment. The AF applied to effect data when deriving risk thresholds is intended to account for uncertainties when extrapolating data from laboratory to field and from model to non-model species. However, the general lack of toxicological effect data for non-model species often makes it necessary to choose the AF without an appropriate knowledge base. For this study, we applied an AF of 10 based on existing guidelines; however, the assembled toxicity data show that inter-species sensitivity can vary by a factor of >100 (SI Figure S2, Table S8). Additional uncertainty arises from the exposure assessment. Analytical constraints, such as limits of quantification that are above effect concentrations, and the selection of analytes, which should be based on usage information and include toxic metabolites, as well as the sampling method and period are important factors to take into consideration. Recent studies by [5,41] showed that the temporal resolution of sampling can significantly influence the results of a risk assessment. MEC detected in grab and composite samples collected over several days or weeks can greatly underestimate short-term peak concentrations of environmental pollutants, especially in small- to medium-sized streams where runoff and flow dynamics tend to be more extreme. This means that acute toxicity is more likely to occur than previously considered. Moreover, resident fish are commonly exposed to multiple stressors including elevated water temperature, changes in the food web and pathogens, which may modulate chemical toxicity [26,27,46,59,72]. The reliance of standard risk assessment approaches on analytical data from grab or composite samples, and on toxicity data derived from laboratory tests with model species and apical endpoints is, therefore, likely to result in an underestimation of the environmental risk of chemicals in Switzerland and elsewhere.

## 4. Conclusions

Our fish-specific risk analysis of pesticide data from monitoring studies conducted in Switzerland showed that pesticides, both as single substance and in mixture, posed a risk for fish at multiple sampling sites. Among hundreds of pesticides analyzed, only three pyrethroid insecticides and two fungicides exceeded fish-specific risk thresholds individually (RQ_i_ ≥ 1). Another eighteen pesticides were detected at lower risk concentrations (RQ_i_ ≥ 0.1). All but five of these relevant pesticides contributed at least 30% to acute or chronic mixture risk in the same water sample and were therefore categorized as highly relevant. Based on available information on sublethal toxicity of highly relevant pesticides, we conclude that fish health may be impaired at environmental concentrations. Sensitive fish species, whose early life stages are present when highest pesticide concentrations occur, are likely to be most vulnerable. However, significant knowledge gaps still exist with regard to the sensitivity of non-model indigenous species, the consequences of non-apical sublethal effects for ecological fitness, the effects of long-term, low-level exposure in combination with intermittent acutely toxic peak pesticide concentrations and the combined effects of multiple stressors on fish health. Current data suggest that such uncertainties are likely to result in an underestimation of environmental risk of pesticides on fish.

Comprehensive long-term monitoring programs together with focused laboratory studies are essential for a better understanding of the ecological risk and impact of chemical pollution. To achieve this, the selection of analytes for characterizing exposure must be checked and revised on a regular basis, as pesticides applied in agriculture change annually depending on weather conditions, crop and regulatory actions. Substances such as the highly relevant pyrethroid insecticides, may have been overlooked by many monitoring programs due to analytical constraints and inadequate limits of quantification. The increased use of bioassays to monitor biological effects rather than individual chemicals could circumvent some of these challenges. However, methods, both analytical and ecotoxicological, to detect chemicals and their effects must continue to be improved utilizing technological advances. Furthermore, methods to extrapolate genomic, ecological and mechanistic information across species may strengthen our understanding of the intrinsic sensitivity of resident species. Eventually, this will lead to better prospective and retrospective risk assessment as well as management to reduce the impact of chemicals in the environment.

## Figures and Tables

**Table 1:** Pesticides posing (A) a single substance risk of RQ_i_ ≥ 1 (acute or chronic); (B) a single substance risk of RQ_i_ ≥ 0.1 and <1 (acute or chronic) in at least one water sample based on monitoring data collected in Switzerland (2012–2018). Substances marked with * contributed at least 30% to mixture risk in the respective sample. Total days of exceedance per monitoring year and number of sites are provided in brackets. IN: insecticide; FU: fungicide; HE: herbicide; -: RQ_i_ < 0.1; NA: substance was not analyzed.

(A) Substances with RQ_i_ ≥ 1		Total Days of Exceedance (Number of Sites Affected)					
		2012	2015		2017 ^a^		2018 ^b^
	RQ_i_	Chronic ^1^	Acute ^2^	Chronic	Acute	Chronic	Chronic
λ-Cyhalothrin * (IN)	≥0.1 ≥1.0	NA	NA	NA	10.5 (1) 3.5 (1)	28 (1) 14 (1)	42 (2)
Cypermethrin * (IN)	≥0.1 ≥1.0	NA	NA	NA	-	14 (1)	42 (1) 14 (1)
Deltamethrin * (IN)	≥0.1 ≥1.0	NA	NA	NA	-	-	28 (1) 14 (1)
Carbendazim * (FU)	≥0.1 ≥1.0	-	62(4)	40.5 (1)	77 (3) 3.5 (1)	66.5 (3)	NA
Fenpropimorph * (FU)	≥0.1 ≥1.0	53 (2)	-	-	-	28 (1) 14 (1)	NA
**(B)** **Substances with RQ_i_ ≥ 0.1 and <1**							
Chlorpyrifos * (IN)	≥0.1	NA	11 (3)	70 (3)	3.5 (1)	63 (3) ^c^	84 (3)
Chlorpyrifos-methyl * (IN)	≥0.1	NA	0.5 (1)	-	-	-	-
Diazinon * (IN)	≥0.1	70 (2)	-	28 (1)	-	-	NA
Permethrin * (IN)	≥0.1	NA	NA	NA	-	-	14 (1)
S-Metolachlor * (HE)	≥0.1	126 (3)	-	56 (1)	-	-	NA
Linuron * (HE)	≥0.1	14 (1)	-	79.5(3)	-	24.5 (1)	NA
Diuron * (HE)	≥0.1	-	-	69.5(1)	-	-	NA
Aclonifen (HE)	≥0.1	-	-	14 (1)	NA	NA	NA
Pendimethalin * (HE)	≥0.1	NA	NA	NA	-	14 (1)	NA
Epoxiconazole * (FU)	≥0.1	42 (2)	-	54.5 (1)	-	52.5 (2)	NA
Tebuconazole (FU)	≥0.1	-	-	-	-	14 (1)	NA
Fluopyram * (FU)	≥0.1	NA	-	40 (1)	-	70 (1)	NA
Flusilazole (FU)	≥0.1	-	-	26.5 (1)	-	14 (1)	NA
Pyraclostrobin * (FU)	≥0.1	28 (1)	-	-	-	-	NA
Spiroxamine * (FU)	≥0.1	-	-	28 (1)	-	-	NA
Azoxystrobin (FU)	≥0.1	-	-	-	-	14 (1)	NA
Fluazinam * (FU)	≥0.1	-	-	-	-	14 (1)	NA
Fludioxonil (FU)	≥0.1	-	-	-	-	14 (1)	NA

^1^ Chronic refers to time-proportional two-week composite samples (2012, 2018) and time-weighted averages over 14 day (2015, 2017), respectively; ^2^ acute refers to: 0.5-day composite samples (up to 24-day during dry periods, 2015) and 3.5-day composite samples (2017); ^a^ pyrethroids were analyzed at only 1 of 5 sites in 2017; ^b^ monitoring in 2018 was focused exclusively on 23 pyrethroid insecticides and chlorpyrifos/-methyl, ^c^ samples collected from one site (Chrümlisbach, BE) in 2017 were additionally analyzed for chlorpyrifos with a lower LOQ as part of a special study; the period of exceedance based on that dataset was 91 days.

## Data Availability

Analytical data used in this study are available at: https://doi.org/10.1021/es500371t (2012), https://doi.org/10.25678/000022 (2015), https://doi.org/10.25678/0000GG (2017) and https://doi.org/10.25678/0001C7 (2018).

## Supplementary Materials

The following are available online at https://www.mdpi.com/article/10.3390/toxics9040079/s1, Table S1: Sources of chemical–analytical data from monitoring studies conducted in Switzerland 2012–2018, Table S2: Acute effect values used in the fish-specific risk assessment (Excel-file: SI_T2), Table S3: Chronic effect values used in the fish-specific risk assessment (Excel-file: SI_T3), Table S4: Acute and chronic single and mixture risk quotients of pesticides detected in water samples of Swiss monitoring studies 2012–2018 (Excel-file: SI_T4 RQmix), Table S5: Effect values, MECs and mode of action of highly relevant substances, Table S6. Sublethal effect data for data-rich relevant pesticides, Table S7: Toxic effect data for data-rich relevant pesticides shown in Figure 3, Table S8: Acute toxicity data (96 h LC50) for data-rich relevant pesticides shown in Figure S2, Table S9: Fish species monitored in small- to medium-sized Swiss streams and their current status, Figure S1: Maximal cumulative ratios (MCR) as a function of the mixture risk quotient of pesticides detected in water samples from monitoring campaigns 2012–2018, Figure S2: Species sensitivity distributions (SSDs) of acute toxicity concentrations for data-rich relevant pesticides. Data for this study is available at https://doi.org/10.25678/00038Y.
