# Supplementary material for: Environmental Risk of Pesticides for Fish in Small- and Medium-Sized Streams of Switzerland
Source: Toxics. 2021 Apr 8;9(4):79. doi: 10.3390/toxics9040079 (PMC8068285; doi:10.3390/toxics9040079)
Supplement: Supplementary file 1 [file toxics-09-00079-s001.zip › Werner_Schneeweiss_et_al_Toxics_2021_SI/Werner_Schneeweiss_et_al_Toxics_2021_SI.pdf]

toxics

MDPI

Supplementary Materials: Environmental Risk of Pesticides for Fish in Small- and Medium-Sized Streams of Switzerland

Inge Werner, Anke Schneeweiss, Helmut Segner and Marion Junghans

SI Table S1. Sources of chemical-analytical data

SI Table S2. Acute effect values used in the fish-specific risk assessment (Excel-file: SI_T_S2)

SI Table S3. Chronic effect values used in the fish-specific risk assessment (Excel-file: SI_T_S3)

SI Table S4. Acute and chronic single substance and mixture risk quotients of pesticides detected in water samples of Swiss monitoring studies 2012-18 (Excel-file: SI_T_S4 RQmix)

SI Table S5. Effect values, MECs and mode of action of highly relevant substances

SI Table S6. Sublethal effect data for data-rich relevant pesticides

SI Table S7. Toxic effect data for data-rich relevant pesticides shown in Figure 3

SI Table S8. Acute toxicity data (96 h LC50) for data-rich relevant pesticides shown in SI Figure S2

SI Table S9. Fish species resident in small to medium-sized streams of Switzerland and their current status

SI Figure S1. Maximal cumulative ratios (MCR) as a function of the mixture risk quotient of pesticides detected in water samples from monitoring campaigns 2012-2018

SI Figure S2. Species sensitivity distributions (SSDs) of acute toxicity concentrations for data-rich relevant pesticides

Table S1. Sources of chemical-analytical data from monitoring studies conducted in Switzerland 2012–18.

Campaign	Reference	Link to data
NAWA 2012	Moschet et al., 2014	https://doi.org/10.1021/es500371t
NAWA 2015	Spycher et al., 2018	https://doi.org/10.25678/000022
NAWA 2017	Spycher et al., 2019	https://doi.org/10.25678/0000GG
Pyrethroids 2017 & 2018	Rösch et al., 2019a, b	https://doi.org/10.25678/0001C7

Table S5. Effect values (in µg/L) for the most sensitive fish species (LC_{50}, NOEC), highest environmental concentration (MEC_{max}; in µg/L) and mode of action of highly relevant pesticides. 1) 14 d composite sample (chronic exposure); 2) 3–3.5 d (acute exposure) composite sample; an assessment factor of 10 was applied to the LC_{50} and NOEC to derive acute and chronic PNECs, respectively. NA: not measured.

Pesticide type	Fish-relevant substances	LC_{50}	NOEC	MEC_{max} 2012[1]	MEC_{max} 2015[2]	MEC_{max} 2015[1]	MEC_{max} 2017[2]	MEC_{max} 2017[1]	MEC_{max} 2018[1]	Primary mode of action
Insecticides	Chlorpyrifos	1.3	0.14	NA	0.039	0.01093	0.021	0.00855	0.0021	Inhibition of acetylcholinesterase
	Chlorpyrifos-methyl	12.6	4.7	NA	0.21	0.00975	0.063	0.01575	0.015	Inhibition of acetylcholinesterase
	Diazinon	90	2.4	0.043	0.59	0.06343	0.0068	0.0068	NA	Inhibition of acetylcholinesterase
	Cypermethrin	0.39	0.03	NA	NA	NA	0.00098	0.00049	0.0038	Disruption of sodium channels
	λ-Cyhalothrin	0.08	0.031	NA	NA	NA	0.031	0.00775	0.00097	Disruption of sodium channels
	Deltamethrin	0.15	0.017	NA	NA	NA	<LOQ	0.00	0.077	Disruption of sodium channels
	Permethrin	1.5	0.66	NA	NA	NA	0.00065	0.00016	0.019	Disruption of sodium channels
Herbicides	Diuron	500	33.4	0.052	3	1.27464	0.093	0.02325	NA	Photosythesis II inhibition
	Linuron	890	21	0.27	2.8	0.748	0.63	0.2295	NA	Photosythesis II inhibition
	S-Metolachlor	3900	30	0.96	5	2.650	0.72	0.2785	NA	Inhibition of long-chain fatty acids
	Pendimethalin	138	6.3	NA	NA	NA	0.48	0.181	NA	Inhibition of mitosis
Fungicides	Carbendazim	7	11	0.065	0.5	0.35214	1.3	0.33648	NA	Damage to cytoskeleton and motor proteins
	Epoxiconazole	3140	3	0.064	0.25	0.05448	0.33	0.10938	NA	Inhibition of sterole biosynthesis
	Fenpropimorph	2300	0.16	0.015	0.0015	0.00046	0.58	0.2	NA	Inhibition of sterole biosynthesis
	Fluazinam	55	2.9	NA	0.024	0.00176	0.23	0.1	NA	Disruption of cellular respiration
	Fluopyram	980	135	NA	6	2.81071	3	1.95	NA	Disruption of cellular respiration
	Pyraclostrobin	6	2	0.0605	0.0045	0.00056	0.0023	0.00105	NA	Disruption of cellular respiration
	Spiroxamine	2410	2	0.016	0.6	0.07507	<LOQ	<LOQ	NA	Inhibition of sterole biosynthesis

Table S6. Sublethal effect data for data-rich relevant pesticides. Data were selected based on environmental relevance in Swiss creeks (≤ MEC_{max} detected in monitoring studies 2012–18). No data was available in this concentration range for S-metolachlor, epoxiconazole, fenpropimorph, fluazinam, fluopyram, flusilazole, pyraclostrobin, spiroxamine and permethrin.

	Substance	*MEC_max*[1] (µg/L)	*LOEC*[2] (µg/L)	Effect (exposure duration)	Fish species and life stage	Reference
Insecticides	Chlorpyrifos	0.039	0.002*	Altered mating behavior in males (1–2 d); reduced number and survival of offspring (14 d)	*Poecilia reticulate*; adult	[1]
			0.005	Reduced AChE[3] activity in head (4 d)	*Odontesthes bonariensis*; larvae	[2]
	Diazinon	0.59	0.0036*	Reduced AChE activity; oxidative stress (5, 15, 30 d)	*Cyprinus carpio*	[3]
			0.3	Altered blood plasma hormone level, reduced level of expressible milt (5 d)	*Salmo salar*; mature males	[4]
			0.47[s]	Reduced fecundity, females (108 d)	*Cyprinodon variegatus*; adult	[5]
			0.55	Reduced growth rate of offspring (6–8 mo)	*Salvelinus fontinalis*; adult	[6]
	Cypermethrin	0.0038	<0.004	Reduced olfactory response to pheromone (PGF2α); Altered blood plasma hormone level, reduced priming effect on milt (5 d)	*Salmo salar*; mature males	[7]
	λ-Cyhalothrin	0.031	0.0004*	DNA damage in erythrocytes (1, 2 d)	*Gambusia affinis*	[8]
			0.005*	Increased hematocrit; DNA damage in erythrocytes; reduced AChE and catalase activity (4 d)	*Prochilodus lineatus*; juvenile	[9]
	Deltamethrin	0.077	0.060	Reduced plasma IgM (28 d)	*Gobiocypris rarus*	[63]
			0.035	Reduced female wet weight in F0 generation (260 d)	*Pimephales promelas ; complete life-cycle*	[64]
			0.049	Reduced dry weight (35 d)	*Cyprinodon variegatus ; eggs (24-48h old)*	[64]
Herbicides	Linuron	2.8	1.0	Reduced vitellogenin concentration in females (21 d)	*Pimephales promelas*; adult	[10]
			1.7	Changes in gene expression (lipid and steroid biosynthesis; cellular stress) (4 d)	*Salmo trutta*; mature males	[11]
	Diuron	3	0.057	Oxidative stress; altered activity of biotransformation enzymes (7 d)	*Oreochromis niloticus*; mature males	[12]
			0.1	Reduction of primary ovarian follicles (25 d)	*Oreochromis niloticus*; mature females	[13]
			0.1	Reduced growth and condition factor; changes in thyroid hormone levels (15 d)	*Menidia beryllina*; juvenile	[14]
			0.2	Reduction in blood plasma testosterone levels (25 d)	*Oreochromis niloticus*; mature males	[15]
			0.2	Changes in gene expression of vitellogenin and activity of cytochrome P450 aromatase (7 d)	*Oreochromis mossambica*; juvenile, male	[16]

			1	Increase of micronuclei in erythrocytes, DNA damage in liver cells and spermatocytes (14, 21 d)	*Danio rerio*; adult, male	[17]
	Pendime-thalin	0.48	0.5	Reduced antioxidant defense in gill and liver (28 d)	*Oncorhynchus mykiss*	[18]
			0.5	Changes in adrenaline level, AChE activity, Na^+/K^+-ATPase activity, monoaminoxidase activity, and oxidative stress (4 d)	*Channa punctata*	[19,20]
**Fungicides**	Carbendazim	0.5	0.16	Changes in swimming behavior (5 d)	*Danio rerio*; larvae	[21]
	Azoxystrobin	3	0.1	Changes in gene expression (oxidative stress, endocrine disruption, immune response) (1, 2, 3 d)	*Danio rerio*; larvae	[22]
			0.25	Changes in anti-oxidative and detoxification enzyme activity/malondialdehyde concentration in liver (7, 14, 21, 28 d)	*Danio rerio*; adult	[23]
			0.5	Increase of micronuclei in erythrocytes, DNA damage in liver cells and spermatocytes (7, 14, 21 d)	*Danio rerio*; adult, male	[17]
			1	Oxidative stress and genotoxicity in liver (7, 14, 21, 28 d)	*Danio rerio*; adult	[24]
			2	Changes in gene expression of *cyp11a, hsd3b, cyp19a* in gonads, *vtg1, vtg2* in liver (21 d)	*Danio rerio*; adult, male	[25]

[1] MEC_{max} from NAWA SPEZ monitoring studies 2015-18; [2] lowest effect concentration; [3] AChE – acetyl cholinesterase; * pesticide applied as product formulation; [S] in saltwater.

Table S7. Toxic effect data for data-rich relevant pesticides shown in Figure 3. LC50: 50% survival after 96 h; NOEC: reduced growth, impaired development or reproduction after ≥28 d; sublethal: non-apical sublethal effects.

Substance	Effect category	Effect concentration (µg/L)	Reference
Linuron	sublethal	1	[10]
Linuron	sublethal	1.7	[11]
Linuron	sublethal	30	[26]
Linuron	sublethal	31.5	[27]
Linuron	sublethal	10	[28]
Linuron	sublethal	30	[29]
Linuron	LC50	2900	[30]
Linuron	LC50	3100	[30]
Linuron	LC50	8600	[30]
Linuron	LC50	9600	[30]
Linuron	LC50	890	[30]
Linuron	NOEC	21	[30]
Diuron	sublethal	0.1	[14]
Diuron	sublethal	78	[31]
Diuron	sublethal	0.057	[12]
Diuron	sublethal	0.2	[15]
Diuron	sublethal	5	[34]
Diuron	sublethal	0.1	[13]
Diuron	LC50	500	[30]
Diuron	LC50	710	[30]
Diuron	LC50	1100	[30]
Diuron	LC50	2800	[30]
Diuron	LC50	6300	[30]
Diuron	LC50	6700	[30]
Diuron	LC50	7700	[30]
Diuron	LC50	7800	[30]
Diuron	LC50	14700	[30]
Diuron	NOEC	33.4	[30]
Diuron	NOEC	220	[30]
Diuron	NOEC	410	[30]
Diazinon	sublethal	0.3	[4]
Diazinon	sublethal	0.47	[5]
Diazinon	sublethal	0.55	[6]
Diazinon	sublethal	2.9	[33]
Diazinon	sublethal	0.0036	[3]
Diazinon	sublethal	45	[34]
Diazinon	sublethal	60	[35]
Diazinon	sublethal	0.675	[36]
Diazinon	sublethal	1	[37]
Diazinon	sublethal	100	[38]
Diazinon	LC50	90	[14]
Diazinon	LC50	168	[30]
Diazinon	LC50	770	[30]
Diazinon	LC50	1470	[30]
Diazinon	LC50	1600	[30]
Diazinon	LC50	4300	[30]
Diazinon	LC50	8900	[30]
Diazinon	NOEC	200	[30]
Diazinon	NOEC	16.5	[30]

Diazinon	NOEC	2.4	[30]
Cypermethrin	sublethal	0.1	[7]
Cypermethrin	sublethal	0.15	[39]
Cypermethrin	sublethal	0.4	[40]
Cypermethrin	sublethal	0.443	[41]
Cypermethrin	sublethal	0.004	[7]
Cypermethrin	sublethal	0.04	[42]
Cypermethrin	sublethal	0.16	[43]
Cypermethrin	LC50	0.4	[30]
Cypermethrin	LC50	0.43	[30]
Cypermethrin	LC50	1.2	[30]
Cypermethrin	LC50	2.1	[30]
Cypermethrin	LC50	1.47	[30]
Cypermethrin	LC50	1.98	[30]
Cypermethrin	LC50	0.71	[30]
Cypermethrin	LC50	1.77	[30]
Cypermethrin	LC50	1.11	[30]
Cypermethrin	LC50	0.39	[30]
Cypermethrin	LC50	3.82	[30]
Cypermethrin	LC50	1.37	[30]
Cypermethrin	LC50	2.4	[30]
Cypermethrin	NOEC	2.79	[30]
Cypermethrin	NOEC	0.03	[30]
Cypermethrin	NOEC	0.077	[30]
Cypermethrin	NOEC	0.37	[30]
Cyhalothrin	sublethal	1	[44]
Cyhalothrin	sublethal	0.0004	[8]
Cyhalothrin	sublethal	0.005	[9]
Cyhalothrin	sublethal	0.15	[45]
Cyhalothrin	sublethal	0.65	[46]
Cyhalothrin	sublethal	0.8	[47]
Cyhalothrin	sublethal	0.3	[48]
Cyhalothrin	LC50	0.078	[30]
Cyhalothrin	LC50	0.16	[30]
Cyhalothrin	LC50	0.21	[30]
Cyhalothrin	LC50	0.24	[30]
Cyhalothrin	LC50	0.49	[30]
Cyhalothrin	LC50	0.78	[30]
Cyhalothrin	LC50	1.6	[30]
Cyhalothrin	LC50	0.7	[30]
Cyhalothrin	LC50	0.807	[30]
Cyhalothrin	NOEC	0.031	[30]
Cyhalothrin	NOEC	0.25	[30]
Chlorpyrifos	sublethal	0.002	[1]
Chlorpyrifos	sublethal	0.12	[49]
Chlorpyrifos	sublethal	0.661	[50]
Chlorpyrifos	sublethal	0.88	[51]
Chlorpyrifos	sublethal	1.2	[52]
Chlorpyrifos	sublethal	0.005	[2]
Chlorpyrifos	sublethal	0.6	[53]
Chlorpyrifos	sublethal	0.625	{54]
Chlorpyrifos	sublethal	1	[55]
Chlorpyrifos	sublethal	0.661	[56]

Chlorpyrifos	sublethal	1.16	[57]
Chlorpyrifos	sublethal	2.25	[58]
Chlorpyrifos	LC50	1.3	[30]
Chlorpyrifos	LC50	1.3	[30]
Chlorpyrifos	LC50	1.7	[30]
Chlorpyrifos	LC50	1.78	[30]
Chlorpyrifos	LC50	4.2	[30]
Chlorpyrifos	LC50	4.7	[30]
Chlorpyrifos	LC50	8.5	[29]
Chlorpyrifos	LC50	8.5	[30]
Chlorpyrifos	LC50	10	[30]
Chlorpyrifos	LC50	12.5	[30]
Chlorpyrifos	LC50	130	[30]
Chlorpyrifos	LC50	136	[30]
Chlorpyrifos	LC50	250	[30]
Chlorpyrifos	LC50	250	[30]
Chlorpyrifos	LC50	520	[30]
Chlorpyrifos	LC50	806	[30]
Chlorpyrifos	NOEC	0.27	[30]
Chlorpyrifos	NOEC	0.75	[30]
Chlorpyrifos	NOEC	0.28	[30]
Chlorpyrifos	NOEC	0.38	[30]
Chlorpyrifos	NOEC	0.51	[30]
Chlorpyrifos	NOEC	5	[30]
Carbendazim	sublethal	4	[59]
Carbendazim	sublethal	20	[59]
Carbendazim	sublethal	0.16	[21]
Carbendazim	LC50	440	[30]
Carbendazim	LC50	1080	[30]
Carbendazim	LC50	7	[30]
Carbendazim	LC50	100	[30]
Carbendazim	LC50	390	[30]
Carbendazim	NOEC	11	[30]
Azoxytrobin	sublethal	0.1	[22]
Azoxytrobin	sublethal	0.25	[23]
Azoxytrobin	sublethal	0.5	[17]
Azoxytrobin	sublethal	1	[24]
Azoxytrobin	sublethal	2	[25]
Azoxytrobin	sublethal	10	[60]
Azoxytrobin	sublethal	20	[61]
Azoxytrobin	sublethal	200	[62]
Azoxytrobin	LC50	470	[30]
Azoxytrobin	LC50	1100	[30]
Azoxytrobin	LC50	1380	[30]
Azoxytrobin	LC50	1600	[30]
Azoxytrobin	LC50	1650	[30]
Azoxytrobin	NOEC	147	[30]

References for SI Tables 6, 7

1. De Silva, P.; Samayawardhena, L. Effects of chlorpyrifos on reproductive performances of guppy *(Poecilia reticulata)*. *Chemosphere* **2005**, *58*, 1293–1299.
2. Aca, V.L.; González, P.V.; Carriquiriborde, P. Lethal and sublethal responses in the fish, Odontesthes bonariensis, exposed to chlorpyrifos alone or under mixtures with endosulfan and lambda-cyhalothrin. *Ecotoxicology* **2018**, *27*, 968–979.
3. Oruç, E.; Usta, D. Evaluation of oxidative stress responses and neurotoxicity potential of diazinon in different tissues of *Cyprinus carpio*. *Environ. Toxicol. Pharmacol*. **2007**, *23*, 48–55.
4. Moore, A.; Waring, C.P. Sublethal effects of the pesticide diazinon on olfactory function in mature male *Atlantic salmon* parr. *J. Fish Biol.* **1996**, *48*, 758–775.
5. Goodman, L.R.; Hansen, D.J.; Coppage, D.L.; Moore, J.C.; Matthews, E. Diazinon: Chronic Toxicity to, and Brain Acetylcholinesterase Inhibition in, the Sheepshead Minnow, *Cyprinodon variegatus*. *Trans. Am. Fish. Soc*. **1979**, *108*, 479–488.
6. Allison, D.T.; Hermanutz, R.O. *Toxicity of Diazinon to Brook Trout and Fathead Minnows*; Environmental Protection Agency, Office of Research and Development: Duluth, MN, USA, 1977.
7. Moore, A.; Waring, C.P. The effects of a synthetic pyrethroid pesticide on some aspects of reproduction in Atlantic salmon *(Salmo salar* L.*)*. *Aquat. Toxicol*. **2001**, *52*, 1–12.
8. Muranli, F.D.G.; Güner, U. Induction of micronuclei and nuclear abnormalities in erythrocytes of mosquito fish *(Gambusia affinis)* following exposure to the pyrethroid insecticide lambda-cyhalothrin. *Mutat. Res. Toxicol. Environ. Mutagen.* **2001**, *726*, 104–108.
9. Vieira, C.E.D.; Dos Reis Martinez, C.B. The pyrethroid λ-cyhalothrin induces biochemical, genotoxic, and physiological alterations in the teleost *Prochilodus lineatus*. *Chemosphere* **2018**, *210*, 958–967.
10. Marlatt, V.L.; Lo, B.P.; Ornostay, A.; Hogan, N.S.; Kennedy, C.J.; Elphick, J.R.; Martyniuk, C.J. The effects of the urea-based herbicide linuron on reproductive endpoints in the fathead minnow *(Pimephales promelas)*. *Comp. Biochem. Physiol. C* **2013**, *157*, 24–32.
11. Webster, T.M.U.; Perry, M.H.; Santos, E.M. The herbicide linuron inhibits cholesterol biosynthesis and induces cellular stress responses in brown trout. *Environ. Sci. Technol*. **2015**, *49*, 3110–3118.
12. Felício, A.A.; Freitas, J.S.; Scarin, J.B.; Ondei, L.D.S.; Teresa, F.B.; Schlenk, D.; de Almeida, E.A. Isolated and mixed effects of diuron and its metabolites on biotransformation enzymes and oxidative stress response of Nile tilapia *(Oreochromis niloticus)*. *Ecotoxicol. Environ. Saf.* **2018**, *149*, 248–256.
13. Pereira, T.S.B.; Boscolo, C.N.P.; Felício, A.A.; Batlouni, S.R.; Schlenk, D.; de Almeida, E.A. Estrogenic activities of diuron metabolites in female Nile tilapia (Oreochromis niloticus). *Chemosphere* **2016**, *146*, 497–502.
14. Moreira, L.B.; Diamante, G.; Giroux, M.; Coffin, S.; Xu, E.G.; Abessa, D.M.D.S.; Schlenk, D. Impacts of Salinity and Temperature on the Thyroidogenic Effects of the Biocide Diuron in *Menidia beryllina*. *Environ. Sci. Technol*. **2018**, *52*, 3146–3155.
15. Pereira, T.S.B.; Boscolo, C.N.P.; da Silva, D.G.H.; Batlouni, S.R.; Schlenk, D.; de Almeida, E.A. Anti-androgenic activities of diuron and its metabolites in male Nile tilapia *(Oreochromis niloticus)*. *Aquat. Toxicol.* **2015**, *164*, 10–15.
16. Felício, A.A.; Crago, J.; Maryoung, L.A.; Almeida, E.A.; Schlenk, D. Effects of alkylphenols on the biotransformation of diuron and enzymes involved in the synthesis and clearance of sex steroids in juvenile male tilapia *(Oreochromus mossambica)*. *Aquat. Toxicol.* **2016**, *180*, 345–352.
17. Bony, S.; Gaillard, I.; Devaux, A. Genotoxicity assessment of two vineyard pesticides in zebrafish. *Int. J. Environ. Anal. Chem.* **2010**, *90*, 421–428.
18. Danion, M.; Le Floch, S.; Lamour, F.; Quentel, C. Effects of in vivo chronic exposure to pendimethalin on EROD activity and antioxidant defenses in rainbow trout *(Oncorhynchus mykiss)*. *Ecotoxicol. Environ. Saf.* **2014**, *99*, 21–27.
19. Tabassum, H.; Afjal, M.A.; Khan, J.; Raisuddin, S.; Parvez, S. Neurotoxicological assessment of pendimethalin in freshwater fish *Channa punctata Bloch*. *Ecol. Indic.* **2015**, *58*, 411–417.
20. Tabassum, H.; Ashafaq, M.; Khan, J.; Shah, Z.; Raisuddin, S.; Parvez, S. Short term exposure of pendimethalin induces biochemical and histological perturbations in liver, kidney and gill of freshwater fish. *Ecol. Indic.* **2016**, *63*, 29–36.
21. Andrade, T.S.; Henriques, J.F.; Almeida, A.R.; Machado, A.L.; Koba, O.; Giang, P.T.; Soares, A.M.; Domingues, I. Carbendazim exposure induces developmental, biochemical and behavioural disturbance in zebrafish embryos. *Aquat. Toxicol*. **2016**, *170*, 390–399.
22. Jiang, J.; Shi, Y.; Yu, R.; Chen, L.; Zhao, X. Biological response of zebrafish after short-term exposure to azoxystrobin. *Chemosphere* **2018**, *202*, 56–64.
23. Jia, W.; Mao, L.; Zhang, L.; Zhang, Y.; Jiang, H. Effects of two strobilurins (azoxystrobin and picoxystrobin) on embryonic development and enzyme activities in juveniles and adult fish livers of zebrafish (*Danio rerio)*. *Chemosphere* **2018**, *207*, 573–580.
24. Han, Y.; Liu, T.; Wang, J.; Wang, J.; Zhang, C.; Zhu, L. Genotoxicity and oxidative stress induced by the fungicide azoxystrobin in zebrafish *(Danio rerio)* livers. *Pestic. Biochem. Physiol*. **2016**, *133*, 13–19.
25. Cao, F.; Zhu, L.; Li, H.; Yu, S.; Wang, C.; Qiu, L. Reproductive toxicity of azoxystrobin to adult zebrafish *(Danio rerio)*. *Environ. Pollut.* **2016**, *219*, 1109–1121.
26. Oulmi, Y.; Braunbeck, T. Cytopathology of liver and in rainbow trout Oncorhynchus mykiss after long-term exposure to sublethal concentrations of linuron. *Dis. Aquat. Org.* **1995**, *21*, 35–52, doi:10.3354/dao021035.
27. Lutnicka, H.; Bartosz Bojarski, B.; Witeska, M.; Tombarkiewicz, B.; Formicki, G. Exposure to herbicide linuron results in alterations in hematological profile and stress biomarkers of common carp (*Cyprinus carpio*). *Ecotoxicology* **2019**, *28*, 69–75, doi:10.1007/s10646-018-2000-y.

28. Tierney, K.B.; Ross, P.S.; Kennedy, C.J. Linuron and carbaryl differentially impair baseline amino acid and bile salt olfactory responses in three salmonids. *Toxicology* **2007**, *231*, 175–187.
29. Topal, A.; Alak, G.; Altun, S.; Erol, H.S.; Atamanalp, M. Evaluation of 8-hydroxy-2-deoxyguanosine and NFkB activation, oxidative stress response, acetylcholinesterase activity, and histopathological changes in rainbow trout brain exposed to linuron. *Environ. Toxicol. Pharmacol.* **2017**, *49*, 14–20.
30. Dossier, Ecotox Centre, Dübendorf, Switzerland. Data Can Be Obtained upon Request from info@oekotoxzentrum.ch. Available online: https://www.oekotoxzentrum.ch (accessed on 7 February 2020.
31. Call, D.J.; Brooke, L.T.; Kent, R.J.; Knuth, M.L.; Poirier, S.H.; Huot, J.M.; Lima, A.R. Bromacil and diuron herbicides: Toxicity, uptake, and elimination in freshwater fish. *Arch. Environ. Contam. Toxicol.* **1987**, *16*, 607–613, doi:10.1007/BF01055817.
32. Saglio, P.; Trijasse, S. Behavioral responses to atrazine and diuron in goldfish. *Arch. Environ. Contam. Toxicol.* **1998**, *35*, 484–491.
33. Flynn, K.; Lothenbach, D.; Whiteman, F.; Hammermeister, D.; Swintek, J.; Etterson, M.; Johnson, R. The effects of continuous diazinon exposure on growth and reproduction in Japanese medaka using a modified Medaka Extended One Generation Reproduction Test (MEOGRT). *Ecotoxicol. Environ. Saf.* **2018**, *162*, 438–445.
34. Dutta, H.M.; Qadri, N.; Ojha, J.; Singh, N.K.; Adhikari, S.; Munshi, J.D.; Roy, P.K. Effect of diazinon on marcophages of bluegill sunfish, Lepomis macrochirus: A cytochemical evaluation. *Bull. Environ. Contam. Toxicol.* **1997**, *58*, 135–141.
35. Banaee, M.; Mir, V.A.; Rafei, G.R.; Majazi, A.B. Effect of sub-lethal diazinon concentrations on blood plasma biochemistry. *Int. J. Environ. Res.* **2008**, *2*, 189–198.
36. Abtahi, B.; Nabavi, H.; Jafari-Shamushaki, V.; Gorbani, R.; Kasumyan, A.O. Influence of insecticides diazinon and endosulfan on taste reception in Persian sturgeon *Acipenser persicus* (Acipenseridae). *J. Appl. Ichthyol.* **2018**, *58*, 248–254.
37. Scholz, N.L.; Truelove, N.K.; French, B.L.; Berejikian, B.A.; Quinn, T.P.; Casillas, E.; Collier, T.K. Diazinon disrupts antipredator and homing behaviors in chinook salmon (*Oncorhynchus tshawytscha*). *Can. J. Fish. Aquat.* **2000**, *57*, 1911–1918.
38. Banaee, M.; Sureda, A.; Mirvaghefi, A.R.; Ahmadi, K. Effects of diazinon on biochemical parameters of blood in rainbow trout (*Oncorhynchus mykiss*). *Pestic. Biochem. Physiol.* **2011**, *99*, 1–6.
39. Poletta, G.L.; Gigena, F.; Loteste, A.; Parma, M.J.; Kleinsorge, E.C.; Simoniello, M.F. Comet assay in gill cells of Prochilodus lineatus exposed in vivo to cypermethrin. *Pestic. Biochem. Physiol.* **2013**, *107*, 385–390.
40. Ansari, R.A.; Rahman, S.; Kaur, M.; Anjum, S.; Raisuddin, S. In vivo cytogenetic and oxidative stress-inducing effects of cypermethrin in freshwater fish, *Channa punctata* Bloch. *Ecotoxicol. Environ. Saf.* **2011**, *74*, 150–156.
41. Vani, T.; Saharan, N.; Roy, S.D.; Ranjan, R.; Pal, A.K.; Siddaiah, G.M.; Kumar, R. Alteration in haematological and biochemical parameters of *Catla catla* exposed to sub-lethal concentration of cypermethrin. *Fish Physiol. Biochem.* **2012**, *38*, 1577–1584.
42. Bonansea, R.I.; Wunderlin, D.A.; Amé, M.V. (2016). Behavioral swimming effects and acetylcholinesterase activity changes in *Jenynsia multidentata* exposed to chlorpyrifos and cypermethrin individually and in mixtures. *Ecotoxicol. Environ. Saf.* **2016**, *129*, 311–319.
43. Sarkar, B.; Chatterjee, A.; Adhikari, S.; Ayyappan, S. Carbofuran-and cypermethrin-induced histopathological alterations in the liver of *Labeo rohita* (Hamilton) and its recovery. *J. Appl. Ichthyol.* **2005**, *21*, 131–135.
44. Tu, W.; Xu, C.; Lu, B.; Lin, C.; Wu, Y.; Liu, W. Acute exposure to synthetic pyrethroids causes bioconcentration and disruption of the hypothalamus–pituitary–thyroid axis in zebrafish embryos. *Sci. Total Environ.* **2016**, *542*, 876–885.
45. Alvim, T.T.; dos Reis Martinez, C.B. Genotoxic and oxidative damage in the freshwater teleost Prochilodus lineatus exposed to the insecticides lambda-cyhalothrin and imidacloprid alone and in combination. *Mutat. Res.-Gen. Toxicol. Environ.* **2019**, *842*, 85–93.
46. Venturini, F.P.; de Moraes, F.D.; Rossi, P.A.; Avilez, I.M.; Shiogiri, N.S.; Moraes, G. A multi-biomarker approach to lambda-cyhalothrin effects on the freshwater teleost matrinxa Brycon amazonicus: Single-pulse exposure and recovery. *Fish Physiol. Biochem.* **2019**, *45*, 341–353.
47. Kumar, A.; Rai, D.K.; Sharma, B.; Pandey, R.S. λ-cyhalothrin and cypermethrin induced in vivo alterations in the activity of acetylcholinesterase in a freshwater fish, *Channa punctatus* (Bloch). *Pestic. Biochem. Physiol.* **2009**, *93*, 96–99.
48. Velmurugan, B.; Selvanayagam, M.; Cengiz, E.I.; Unlu, E. Histopathology of lambda-cyhalothrin on tissues (gill, kidney, liver and intestine) of *Cirrhinus mrigala*. *Environ. Toxicol. Pharmacol.* **2007**, *24*, 286–291.
49. Jarvinen, A.W.; Nordling, B.R.; Henry, M.E. Chronic toxicity of Dursban (chlorpyrifos) to the fathead minnow (*Pimephales promelas*) and the resultant acetylcholinesterase inhibition. *Ecotoxicol. Environ. Saf.* **1983**, *7*, 423–434.
50. Raibeemol, K.P.; Chitra, K.C. Effects of chlorpyrifos as inducer for oxidative stress in liver, kidney and spleen of freshwater fish, *Pseudetroplus maculatus* (Bloch, 1795). *Res. Rev. J. Toxicol.* **2018**, *8*, 20–29.
51. Marchand, A.; Porcher, J.-M.; Turies, C.; Chadili, E.; Palluel, O.; Baudoin, P.; Betoulle, S.; Bado-Nilles, A. Evaluation of chlorpyrifos effects, alone and combined with lipopolysaccharide stress, on DNA integrity and immune responses of the three-spined stickleback, *Gasterosteus aculeatus*. *Ecotoxicol. Environ. Saf.* **2017**, *145*, 333–339.
52. Eder, K.J.; Leutenegger, C.M.; Wilson, B.W.; Werner, I. Molecular and cellular biomarker responses to pesticide exposure in juvenile Chinook salmon (*Oncorhynchus tshawytscha*). *Mar. Environ. Res.* **2004**, *58*, 809–813.
53. Sandahl, J.F.; Baldwin, D.H.; Jenkins, J.J.; Scholz, N.L. Comparative thresholds for acetylcholinesterase inhibition and behavioral impairment in coho salmon exposed to chlorpyrifos. *Environ. Toxicol. Chem.* **2005**, *24*, 136–145, doi:10.1897/04-195r.1.
54. Sandahl, J.F.; Baldwin, D.H.; Jenkins, J.J.; Scholz, N.L. Odor-evoked field potentials as indicators of sublethal neurotoxicity in juvenile coho salmon (*Oncorhynchus kisutch*) exposed to copper, chlorpyrifos, or esfenvalerate. *Can. J. Fish. Aquat. Sci.* **2004**, *61*, 404–413.

55. Hayman, N.T.; Hentschel, B.T.; Renick, V.C.; Anderson, T.W. Combined effects of flow speed and sub-lethal insecticide exposure on predator–prey interactions between the California killifish and an infaunal polychaete. *Ecotoxicology* **2019**, *28*, 117–131, doi:10.1007/s10646-018-2005-6.
56. Raibeemol, K.P.; Chitra, K.C. Hematological and biochemical changes in the freshwater fish, Pseudetroplus maculatus exposed to sublethal concentrations of chlorpyrifos. *Res. Rev.* **2018**, *8*, 108–116.
57. Xing, H.; Li, S.; Wang, Z.; Gao, X.; Xu, S.; Wang, X. Oxidative stress response and histopathological changes due to atrazine and chlorpyrifos exposure in common carp. *Pestic. Biochem. Physiol.* **2012**, *103*, 74–80.
58. Topal, A., Atamanalp, M., Oruç, E., Demir, Y., Beydemir, Ş., Işık, A. In vivo changes in carbonic anhydrase activity and histopathology of gill and liver tissues after acute exposure to chlorpyrifos in rainbow trout. Arch. Ind. Hyg. Toxicol., **2014**, *65*, 377385.
59. Jiang, J.; Wu, S.; Wang, Y.; An, X.; Cai, L.; Zhao, X.; Wu, C. Carbendazim has the potential to induce oxidative stress, apoptosis, immunotoxicity and endocrine disruption during zebrafish larvae development. *Toxicology In Vitro* **2015**, *29*, 1473–1481.
60. Liu, L.; Jiang, C.; Wu, Z.Q.; Gong, Y.X.; Wang, G.X. Toxic effects of three strobilurins (trifloxystrobin, azoxystrobin and kresoxim-methyl) on mRNA expression and antioxidant enzymes in grass carp (*Ctenopharyngodon idella*) juveniles. *Ecotoxicol. Environ. Saf.* **2013**, *98*, 297–302.
61. Cao, F.; Li, H.; Zhao, F.; Wu, P.; Qian, L.; Huang, L.; Pang, S.; Martyniuk, C.J.; Qiu, L. Parental exposure to azoxystrobin causes developmental effects and disrupts gene expression in F1 embryonic zebrafish (*Danio rerio*). *Sci. Total Environ.* **2019**, *646*, 595–605.
62. Cao, F.; Wu, P.; Huang, L.; Li, H.; Qian, L.; Pang, S.; Qiu, L. Short-term developmental effects and potential mechanisms of azoxystrobin in larval and adult zebrafish (*Danio rerio*). *Aquat. Toxicol.* **2018**, *198*, 129–140.
63. Zhang, L.; Hong, X.; Zhao, X.; Yan, S.; Ma, X.; Zha, J. Exposure to environmentally relevant concentrations of deltamethrin renders the Chinese rare minnow (*Gobiocypris rarus*) vulnerable to Pseudomonas fluorescens infection. *Sci. Total Environ.* **2020**, *715*, 136943, doi:10.1016/j.scitotenv.2020.136943.
64. *Draft Renewal Assessment Report (DRAR) prepared according to the Commission Regulation (EU) N° 1107/2009—DELTAMETHRIN*; European Commission (EC): Rapporteur, UK; Co-Rapporteur, Austria, 2018; Volume 3, B.9(AS), pp. 66–67.

Table S8. Acute toxicity data (96 h LC50) for data-rich relevant pesticides shown in SI Figure S2. All data were examined for validity and relevance by the Ecotox Centre, Dübendorf, Switzerland (https://www.oekotoxzentrum.ch) and can be obtained upon request from info@oekotoxzentrum.ch.

Substance	LC50 (µg/L)	Fish species	Family	Order
azoxystrobin	470	*Oncorhynchus mykiss*	Salmonidae	Salmoniformes
azoxystrobin	1100	*Lepomis macrochirus*	Centrarchidae	Perciformes
azoxystrobin	1380	*Oryzias latipes*	Adrianichthyidae	Beloniformes
azoxystrobin	1600	*Cyprinus carpio*	Cyprinidae	Cypriniformes
azoxystrobin	1650	*Misgurnus anguilicaudatus*	Cobitidae	Cypriniformes
azoxystrobin	660	*Cyprinodon variegatus*	Cyprinodontidae	Cyprinodontiformes
carbendazim	440	*Cyprinus carpio*	Cyprinidae	Cypriniformes
carbendazim	1080	*Danio rerio*	Cyprinidae	Cypriniformes
carbendazim	7	*Ictalurus punctatus*	Ictaluridae	Siluriformes
carbendazim	100	*Oncorhynchus mykiss*	Salmonidae	Salmoniformes
carbendazim	390	*Salmo trutta*	Salmonidae	Salmoniformes
chlorpyrifos	1.3	*Leuresthes tenuis*	Atherinopsidae	Atheriniformes
chlorpyrifos	1.3	*Menidia peninsulae*	Atherinopsidae	Atheriniformes
chlorpyrifos	1.7	*Menidia menidia*	Atherinopsidae	Atheriniformes
chlorpyrifos	1.78	*Lepomis macrochirus*	Centrarchidae	Perciformes
chlorpyrifos	4.2	*Menidia beryllina*	Atherinopsidae	Atheriniformes
chlorpyrifos	4.7	*Pungitius pungitius*	Gasterosteidae	Gasterosteiformes
chlorpyrifos	8.5	*Oncorhynchus mykiss*	Salmonidae	Salmoniformes
chlorpyrifos	8.5	*Gasterosteus aculeatus*	Gasterosteidae	Gasterosteiformes
chlorpyrifos	10	*Leuciscus Idus*	Cyprinidae	Cypriniformes
chlorpyrifos	12.5	*Sander vitreus*	Percidae	Perciformes
chlorpyrifos	130	*Pimephales promelas*	Cyprinidae	Cypriniformes
chlorpyrifos	136	*Cyprinidon variegatus*	Cyprinodontidae	Cyprinodontiformes
chlorpyrifos	250	*Oryzias latipes*	Adrianichthyidae	Beloniformes
chlorpyrifos	250	*Rutilus rulitus*	Cyprinidae	Cypriniformes
chlorpyrifos	520	*Opsanus beta*	Batrachoididae	Batrachoidiformes
chlorpyrifos	806	*Ictalurus punctatus*	Ictaluridae	Siluriformes
cyhalothrin	0.078	*Leuciscus Idus*	Cyprinidae	Cypriniformes
cyhalothrin	0.16	*Ictalurus punctatus*	Ictaluridae	Siluriformes
cyhalothrin	0.21	*Lepomis macrochirus*	Centrarchidae	Perciformes
cyhalothrin	0.24	*Oncorhynchus mykiss*	Salmonidae	Salmoniformes
cyhalothrin	0.49	*Gasterosteus aculeatus*	Gasterosteidae	Gasterosteiformes
cyhalothrin	0.78	*Danio rerio*	Cyprinidae	Cypriniformes
cyhalothrin	1.6	*Oryzias latipes*	Adrianichthyidae	Beloniformes
cyhalothrin	0.7	*Pimephales promelas*	Cyprinidae	Cypriniformes
cyhalothrin	0.807	*Cyprinodon variegatus*	Cyprinodontidae	Cyprinodontiformes
cypermethrin	0.4	*Scardinius erythrophthalmus*	Cyprinidae	Cypriniformes
cypermethrin	0.43	*Cnesterodon decemmaculatus*	Poecilidae	Cyprinodontiformes
cypermethrin	1.2	*Salmo trutta*	Salmonidae	Salmoniformes
cypermethrin	2.1	*Oreocchromis niloticus*	Cichlidae	Cichliformes
cypermethrin	1.47	*Galaxius maculatus*	Galaxiidae	Osmeriformes
cypermethrin	1.98	*Pseudaphritis urvillii*	Pseudaphritidae	Perciformes
cypermethrin	0.71	*Cyprinus carpio*	Cyprinidae	Cypriniformes
cypermethrin	1.77	*Brachidanio rerio*	Cyprinidae	Cypriniformes
cypermethrin	1.11	*Lepomis macrochirus*	Centrarchidae	Perciformes
cypermethrin	0.39	*Oncorhynchus mykiss*	Salmonidae	Salmoniformes
cypermethrin	3.82	*Oryzias latipes*	Adrianichthyidae	Beloniformes
cypermethrin	1.37	*Pimephales promelas*	Cyprinidae	Cypriniformes
cypermethrin	2.4	*Cyprinodon variegatus*	Cyprinodontidae	Cyprinodontiformes
diazinon	90	*Oncorhynchus mykiss*	Salmonidae	Salmoniformes
diazinon	168	*Lepomis macrochirus*	Centrarchidae	Perciformes
diazinon	770	*Salvelinus fontinalis*	Salmonidae	Salmoniformes
diazinon	1470	*Cyprinidon variegatus*	Cyprinodontidae	Cyprinodontiformes
diazinon	1600	*Jordanella floridae*	Cyprinodontidae	Cyprinodontiformes
diazinon	4300	*Pimephales promelas*	Cyprinidae	Cypriniformes

diazinon	8900	*Entosphenus tridentatus*	Petromyzontidae	Petromyzontiformes
diuron	500	*Morone saxatilis*	Moronidae	Perciformes
diuron	710	*Oncorhynchus clarki*	Salmonidae	Salmoniformes
diuron	1100	*Salvelinus namaycush*	Salmonidae	Salmoniformes
diuron	2800	*Lepomis macrochirus*	Centrarchidae	Perciformes
diuron	6300	*Mugil cephalus*	Mugilidae	Mugiliformes
diuron	6700	*Cyprinodon variegatus*	Cyprinodontidae	Cyprinodontiformes
diuron	7700	*Pimaphales promelas*	Cyprinidae	Cypriniformes
diuron	7800	*Oryzias melastigma*	Adrianichthyidae	Beloniformes
diuron	14700	*Oncorhynchus mykiss*	Salmonidae	Salmoniformes

Table S9. Fish species resident in small to medium-sized streams in Switzerland and their current status (NAWA TREND Monitoring [1,2]).

**Species**	**Red List Status**
Chondrostoma nasus	Critically endangered
Salmo trutta lacustris	Endangered
Lampetra planeri	Endangered
Thymallus thymallus	Vulnerable
Alburnoides bipunctatus	Vulnerable
Leuciscus souffia	Vulnerable
Cyprinus carpio	Vulnerable
Anguilla anguilla	Vulnerable
Cobitis taenia	Vulnerable
Salmo trutta fario	Near threatened
Gasterosteus aculeatus	Near threatened
Cottus gobio	Near threatened
Barbus barbus	Near threatened
Perca fluviatilis	Least concern
Esox lucius	Least concern
Leuciscus cephalus	Least concern
Leuciscus leuciscus	Least concern
Rutilus rutilus	Least concern
Scardinius erythrophthalmus	Least concern
Tinca tinca	Least concern
Lota lota	Least concern
Gobio gobio	Least concern
Phoxinus phoxinus	Least concern
Barbatula barbartula	Least concern
Gymnocephalus cernuus	Least concern

1 Kirchhofer, A.; Breitenstein, M.; Zaugg, B. Rote Liste der Fische und Rundmauler der Schweiz. Umwelt-Vollzug Nr. 0734; Bundesamt für Umwelt, Bern, und Schweizer Zentrum für die Kartographie der Fauna, Neuenburg, Switzerland.**2007**; p. 64.
2 Spalinger, L.; Dönni, W.; Guthruf, J. *NAWA TREND BIOLOGIE 2. Kampagne (2015) Fachbericht Fische, Report*; Bundesamt für Umwelt: Bern, Switzerland, **2017**; p. 75.

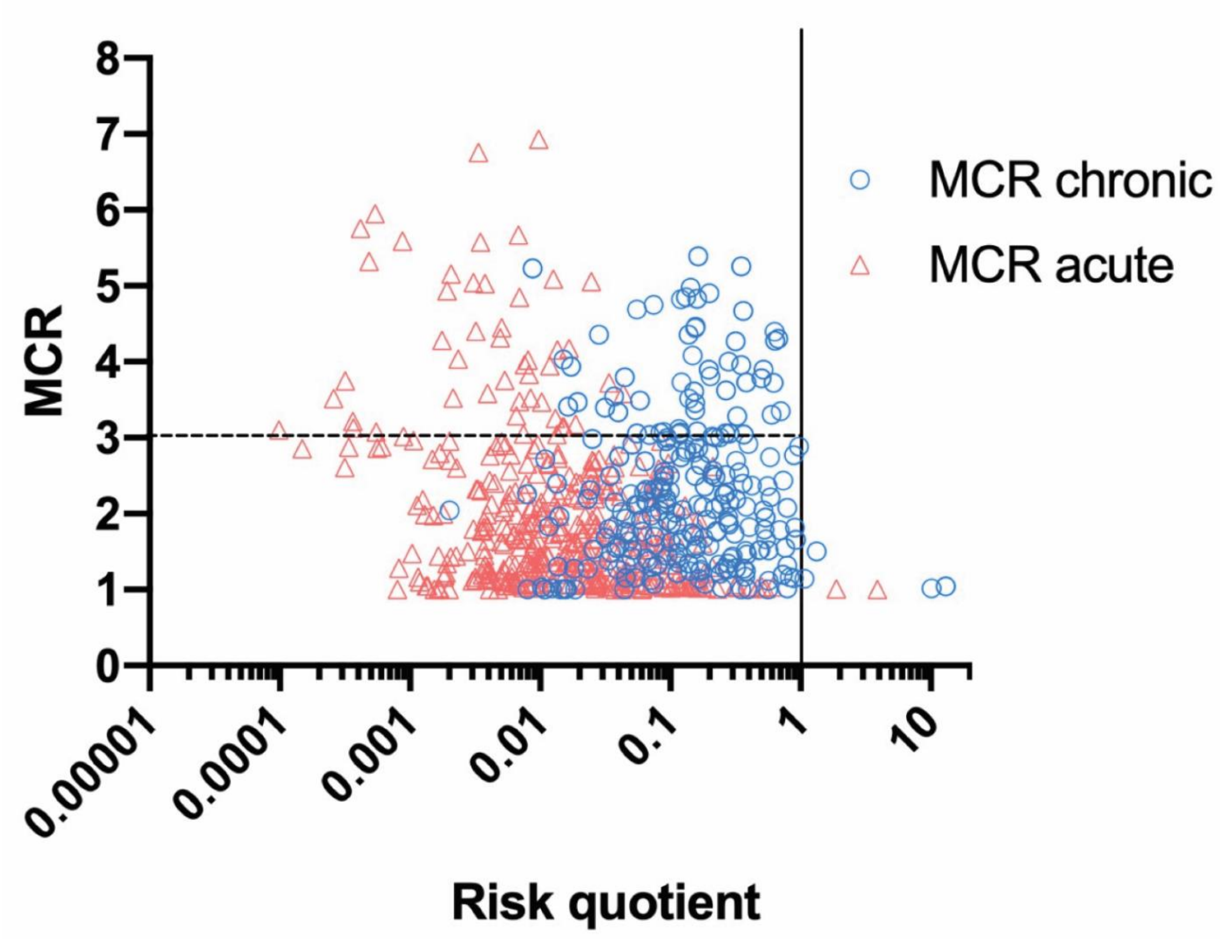

Figure S1. Maximum cumulative ratios (MCR) as a function of the mixture risk quotient of pesticides detected in water samples from monitoring campaigns 2012-2018. The MCR corresponds to the number of pesticides in the sample that drive the mixture risk quotient.

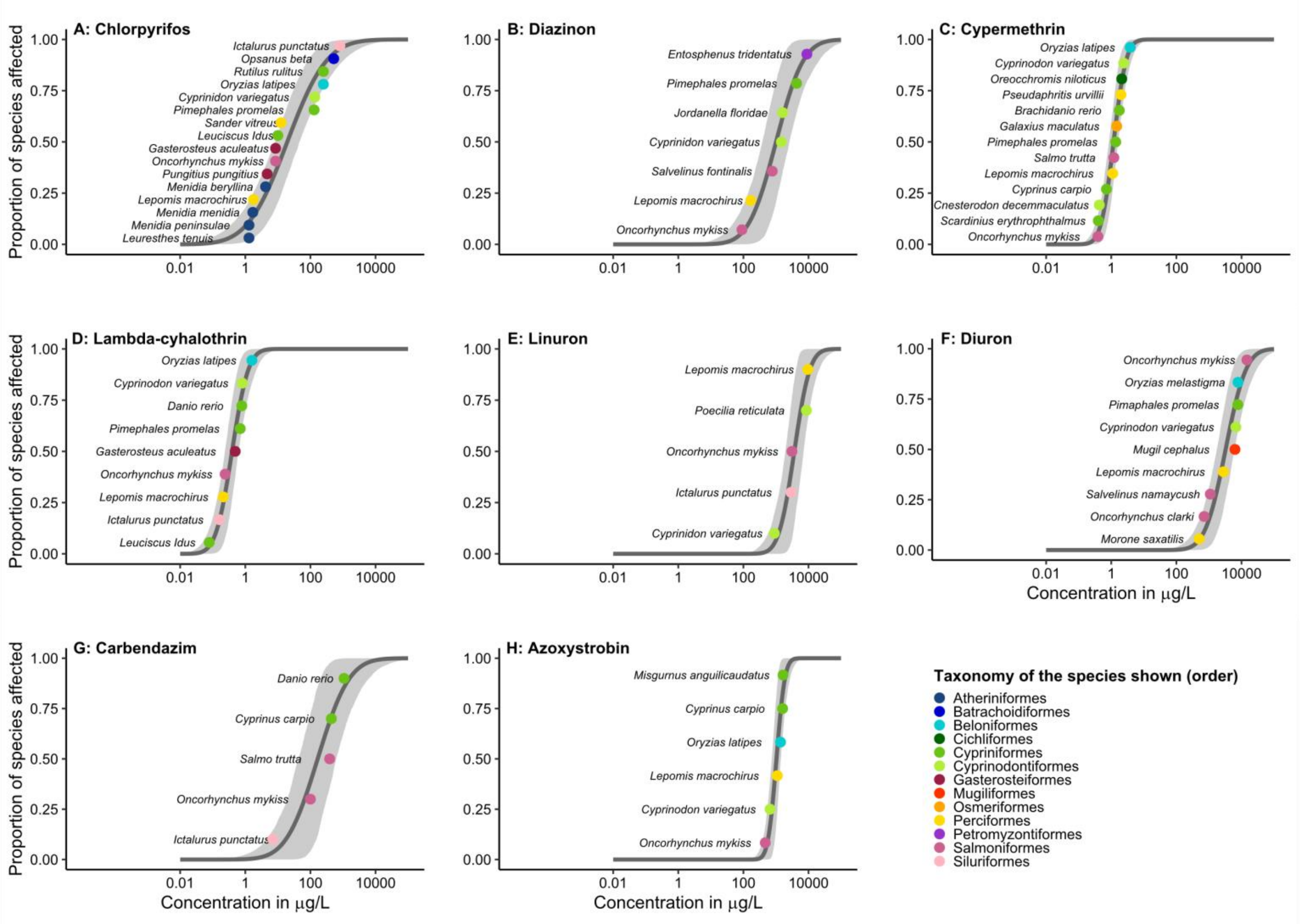

Figure S2. Species sensitivity distributions (SSDs) for data-rich relevant pesticides.
